# Medical student perceptions of mental illness: a cross-sectional transnational study in two medical schools

**DOI:** 10.1186/s12909-023-04962-2

**Published:** 2023-12-20

**Authors:** Annie Rees, Callum Cuthbert, Viraj Shah, Lim Rong, Daniel Peh, Ana Baptista, Susan Smith

**Affiliations:** 1 School of Medicine, Imperial College London, London, UK; 2 Lee Kong Chian School of Medicine, Nanyang Technological University, Singapore, Singapore; 3 Medical Education Research Unit, Faculty of Medicine, Imperial College London, London, UK; 4 National Heart and Lung Institute, Imperial College London, London, UK

**Keywords:** Stigma, Mental health, Mental illness, Medical education, Medical curriculum, International

## Abstract

**Background:**

Despite shifting global attitudes, mental illness remains highly stigmatised amongst practicing doctors. This has wider implications on doctors’ training to care for patients with mental illness. There is need for exploration of the presence and mitigation of stigma in early medical education to prevent such attitudes propagating into clinical practice. Thus, this study explores whether stigmatising attitudes are detectable amongst medical students in London and Singapore and examines whether they are ameliorated by specific curricular and welfare features of formal medical education, utilising the Mental Illness Stigma Framework (MISF).

**Methods:**

A mixed-methods approach was adopted. Medical students at Imperial College London (UK; n = 211) and Nanyang Technological University (Singapore; n = 141) completed a validated scale (the OMS-HC-15) to assess attitudes towards mental illness. Semi-structured interviews were conducted (Imperial: n = 12, NTU: n = 8) until theoretical saturation was reached. Quantitative data were analysed descriptively and comparatively using SPSS and interview data subjected to inductive thematic analysis.

**Results:**

Total OMS-HC-15 scores ranged from 19–51 for Imperial (n = 211) and 16–53 for NTU (n = 141). No significant differences in overall stigma scores were found between the two schools (p = 0.24), nor when comparing year groups within each school. Four themes were identified across interview data: student perceptions, impacts of medical school culture, university support, and curricular impacts on mental illness perceptions. Themes allowed identification of aspects of medical school that were well-received and warranted further emphasis by students, alongside areas for improvement.

**Conclusion:**

Mental health stigma was identified in two medical schools, with differing cultures. Mean stigma scores obtained were comparable between both UK and Singaporean medical students. Nuanced differences were identified via subgroup analysis, and the MISF identified both shared and country-specific drivers for this stigma across the qualitative data. Actionable recommendations to mitigate this were hypothesised. Curricular improvements such as earlier psychiatric teaching and sharing of personal stories may improve future stigma scores as students’ progress through the course. Specific welfare-based changes to formal support systems were also deemed to be beneficial by students. The impacts of welfare and curricular redesign in relation to societal influence on students’ attitudes warrants further investigation, as does medical students’ self-stigma.

**Supplementary Information:**

The online version contains supplementary material available at 10.1186/s12909-023-04962-2.

## Background

Recent years have seen paradigm shifts in global attitudes towards mental health [1]. Greater public awareness, through education and media campaigns [2], has resulted in sociocultural discussions regarding mental health globally [3]. Despite these efforts, stigmatising attitudes remain prevalent amongst doctors, resulting in insufficient preparation to care for patients with mental health issues [4].

The concept of ‘stigma’ remains complex with varying definitions. However, common underpinning factors include labelling, discrimination, and stereotyping [5]. One influential definition is that of Jones et al. [6], proposing that stigma is a ‘mark’ that links a person to undesirable characteristics; this was conceptualised from Goffman’s [7] earlier observation of stigma as a relationship between an ‘attribute and a stereotype’.

Stigma includes self, public, and institutional components [8]. Self-stigma of those with mental illness has multifactorial socioecological effects such as decreased standard of living, fewer employment opportunities, and poor self-esteem [9]. Public and institutional stigma shape that of the self as individuals avoid labelling and stereotyping [8]. Health care providers (HCPs) continue to harbour stigma towards patients with mental illness [10–12] thus negatively shaping health outcomes as individuals with mental illness experience barriers to receiving high-quality healthcare [4, 13].

Whilst some papers identify stigma amongst medical students, they rarely explore the underlying causes, nor provide recommendations to address said stigma [14]. When recommendations are given, they usually lack a qualitative basis [15] and do not come from students themselves, and rarely compare countries with disparate cultures [16], and hence lack a framework that may be applied transnationally.

The Mental Illness Stigma Framework (MISF) [17] breaks down societal stigma to that of the stigmatised (e.g. the mentally ill patient) and the stigmatiser (e.g. the HCP). Perspectives of the stigmatiser are underpinned by stereotyping, discrimination, and prejudice, whereas the stigmatised explores internalised, anticipated, and experienced stigma resulting in delayed treatment seeking, poor treatment adherence, worsened mental health and social well-being. Contributing intersectional characteristics include culture, race, gender, and sexual orientation.

Wallace [12] places the culture of medical training as pivotal to addressing prejudice displayed by HCPs. Studies performed in various countries identify stigmatising attitudes within medical students [14–16], highlighting that early intervention within medical training is vital and opportune, and that whilst cultural views of mental health vary from country to country, this remains a global issue for undergraduate teaching. Educational projects in the United Kingdom (UK) have targeted medical trainees, notably the Education Not Discrimination anti-stigma course [18] by the UK-based Time to Change initiative, however, they have only offered short-term stigma reduction [19–21].

The UK and Singapore are two countries that have shown commitment in recent years to improving outcomes for mentally ill individuals. The Department of Health in the UK proposed a ten-year mental health strategy in 2021 which focusses on the national roll-out of mental health hubs to increase accessibility for early intervention and prevention of mental illnesses [22]. Further efforts shown include Public Health England’s Every Mind Matters social media campaign, aiming to improve mental health literacy within the UK [23]. Singapore’s Institute of Mental Health has similarly shown recent efforts to improve mental health literacy via their HOPE intervention [24], aimed particularly at undergraduates, as well as their national Mind Matters mental health literacy study 2022–23 [25]. Currently there is a lack of research exploring the effect of national and institutional culture on stigma and their intersectionality with medical education.

Our study aimed to identify whether stigmatising attitudes were present amongst medical students from two institutions, one from each country: Imperial College London (Imperial)(UK) and Nanyang Technological University (NTU)(Singapore). These two countries were selected due to their aforementioned interest in advancing mental health outcomes, as well as to compare the intersectional effect of how culture and race affect stigma, as per the MISF. The NTU student authors of this manuscript will receive degrees jointly awarded by Imperial and NTU, although lower years have diverged. Each curriculum follows a standard MBBS structure: two pre-clinical years followed by three years of predominant clinical placements with teaching. Of note, Imperial changed the undergraduate curriculum from 2019 [26]—see Table 1 for differences between the two curricula used. The new curriculum integrates increased clinical exposure in the earlier years of the course. Imperial has an additional compulsory intercalated year. Further study aims included identifying differences between stigmatising attitudes and understanding underpinning factors causing these disparities, with a particular focus on the curriculum and medical school culture and how these may inform interventions to better address mental illness stigma in undergraduate medical education.

**Table 1:** Differences in mental health teaching between imperial old and new curricula

Year	Old Curricula Differences	New Curricula Differences	Similarities
1	“Neuroscience and Mental Health Module” – Lectures teaching about Dementia and Depression	Replacement of “Neuroscience and Mental Health Module” with “Psychiatry” module– Lectures, tutorials and workshops on a broader range of presentations (addition of anxiety, bipolar disorder, OCD, eating disorders, self-harm, addiction and schizophrenia) Increased emphasis on biopsychosocial care in redesigned “Patients, Communities & Healthcare” and “Professional Values & Behaviour” modules Novel “Lifestyle Medicine & Prevention” module, with content on mental health and student health Increased integrated student wellbeing teaching	Compulsory lectures and assessment on the presentation of dementia conditions and depression
2	“Neuroscience and Mental Health Module” – Lectures teaching about Dementia and Depression	Replacement of “Neuroscience and Mental Health Module” with “Psychiatry” module– Lectures, tutorials and workshops building on Year 1 content with more detail and depth of understanding (addition of lifecourse psychiatry and psychopharmacology) Continued emphasis on biopsychosocial care in redesigned “Patients, Communities & Healthcare” and “Professional Values & Behaviour” modules Novel “Lifestyle Medicine & Prevention” module, with content on mental health and student health Increased integrated student wellbeing teaching	Compulsory lectures and assessment on the presentation of dementia conditions and depression
3	Inclusion of Depression and Dementia conditions within yearly Learning Objectives	Expansion of ‘Depression’ and ‘Dementia’ conditions included within Learning Objectives to add ‘Delirium’, ‘Wernicke’s encephalopathy’ and ‘Generalised Anxiety Disorder’ Inclusion of learning objectives on holistic presentations with a mental health component including ‘Sleep problems’, ‘Overdose’, ‘Appetite change’, ‘Weight gain’, ‘Palpitations’, ‘Low mood and affective problems’, ‘Headache’, ‘Alcoholic hepatitis’, ‘Subdural bleed’, ‘Change in libido’, ‘Elder abuse’, ‘Hallucinations’, ‘Terminal illness’, ‘Struggling to cope’, ‘Confusion’, ‘Memory loss’, ‘Mental capacity concerns’, ‘Abdominal pain’ and ‘Driving advice’	Assessment on the presentation of dementia conditions and depression
4	N/A (no difference)	N/A (no difference)	Optional Neuroscience and Mental Health Intercalated BSc degree
5	8-week psychiatry placement with patient contact and weekly lectures on mental health conditions, including stigma workshop	Redesigned 6-week psychiatry placement with patient contact and weekly lectures on mental health conditions, including stigma workshop as well as digital psychiatry teaching, with greater focus on patient stories and diversity of psychiatry encounters	Minimum 6 weeks of patient contact in clinical psychiatry environment, weekly mental health lectures, and stigma workshop
6	N/A (no difference)	N/A (no difference)	No MH-specific teaching in Y6

## Methods

This study employed a mixed methods design whereby surveys were followed with semi-structured interviews (based on questionnaire responses). Survey data provided quantitative comparisons of students at each medical school to identify any differences in attitudes towards mental illness. Interviews allowed deeper exploration of these attitudes, providing suggestions on ways to better address mental health stigma.

### Recruitment

Invitations to complete the survey were circulated via social media and official medical school communication streams. Medical students across all years at both schools were eligible to participate with recruitment occurring September—December 2021. On completion of the survey, participants were provided a link to express interest in undertaking an optional individual interview. Two volunteers were then randomly selected from each year group from each school. A sample size calculator was applied to the total student population at Imperial and NTU to identify a minimum sample size of 158 and 139 respectively with a 95% confidence level and 7.5% margin of error. Supplementarily, similar international literature achieved sample sizes ranging from 102–265 [27, 28], affirming confidence in the calculated minimum sample size.

### Quantitative data collection

The survey consisted of the validated Opening Minds Stigma Scale for Healthcare Providers (OMS-HC-15) scale [27] and basic demographic questions. The OMS-HC-15 is a self-report questionnaire assessing attitudes of HCPs towards people with mental illness. Overall scores range from 15 (least stigma held) to 75 (most stigma held). Three subscales considered different aspects of stigma: Subscale 1 *(Attitudes of healthcare providers towards people with mental illness)* ranging from 6–30, Subscale 2 *(Attitudes of healthcare providers towards disclosure and help-seeking)* ranging from 4–20 and Subscale 3 *(Attitudes of healthcare providers towards social distance)* ranging from 5–25. In all scales, the lower the score, the less stigmatising the attitudes. Subscales 1 and 3 explore the view of the stigmatiser, whilst subscale 2 incorporates that of the stigmatised, in relation to the MISF. See Appendix 1 for more detail of the scale and subscales.

### Qualitative data collection

We developed interview questions (Appendix 2) based on survey results to explore how the respective medical schools shape students’ perceptions of mental illness. Interviews were semi-structured. Questions explored effects of the intersectional characteristics of race and culture as well as the roles of the stigmatiser, in terms of the individual, faculty and other students, and the role of the stigmatised, where appropriate. All interviews were conducted by two members of the study team, from the opposite school to the participant. Interviews lasted 30–40 min. Participants provided written and oral informed consent prior to the interview. Interviews were conducted over Zoom and audio recorded. Recordings were anonymised, manually transcribed (V.S., A.R., C.C., L.R., and D.P.), and stored on an encrypted server.

### Data analysis

Quantitative data were analysed using non-parametric, Mann–Whitney U and Kruskal–Wallis tests using IBM SPSS Statistics 26.0 (SPSS Inc., Chicago, Ill., USA); figures were created with GraphPad Prism 9 (GraphPad Software, Inc., San Diego, CA, USA). Qualitative data were analysed using basic thematic analysis [28], using NVivo 12 (QSR International, Pty Ltd, Australia). A codebook was developed from initial impressions of the data and from a literature review. Each transcript was systematically coded by one study member, then reviewed by a second. Study members collectively identified overarching themes from coded data applicable to the study aims.

### Participant demographics

The questionnaire was disseminated to all students within the medical schools at Imperial (N = 2200) and NTU (N = 736). 352 students completed the survey (Imperial: n = 211; NTU: n = 141). Participant demographics are shown in Table 2. 20 students were interviewed in total [Imperial: n = 12 (Years 1–6); NTU: n = 8 (Years 1–4)]. NTU Year 5 students contributed to the survey data but were unable to participate in the interview stage due to their upcoming examinations.

**Table 2:** Participant demographic data split via year group, gender, sexuality, ethnicity, and previous mental illness

	**Imperial**	**NTU**	**Total**
**Year Group**			
Year 1	50	25	**75 (21.3%)**
Year 2	51	49	**100 (28.4%)**
Year 3	29	19	**48 (13.6%)**
Year 4	22	28	**50 (14.2%)**
Year 5	29	20	**52 (14.8%)**
Year 6	30	-	**30 (8.5%)**
**Gender**			
Cisgender male	68	74	**142 (40.3%)**
Cisgender female	138	63	**201 (57.1%)**
Other	2	1	**3 (0.9%)**
Prefer not to say	3	3	**6 (1.7%)**
**Sexuality**			
Heterosexual	158	120	**278 (79.0%)**
Homosexual (Gay or Lesbian)	6	1	**7 (2.0%)**
Bisexual	30	6	**36 (10.2%)**
Other	10	1	**11 (3.1%**
Prefer not to say	7	13	**20 (5.7%)**
**Ethnicity**			
White British or Irish	40	0	**40 (11.4%)**
White Other	16	0	**16 (4.5%)**
Black, African, Caribbean, Black British	7	0	**7 (2.0%)**
Asian/Asian British (Chinese)	27	125	**152 (43.2%)**
Asian/Asian British (Indian, Pakistani)	62	9	**71 (20.2%)**
Asian/Asian British (Bangladeshi)	10	0	**10 (2.8%)**
Any Other Asian	16	2	**18 (5.1%)**
Arab	5	0	**5 (1.4%)**
Mixed or Multiple Ethnicities	12	2	**14 (4.0%)**
Any Other Ethnic Groups	9	1	**10 (2.8%)**
Prefer not to say	7	2	**9 (2.6%)**
**History of mental illness**			
Yes	79	18	**97 (27.6%)**
No	120	114	**234 (66.5%)**
Prefer not to say	12	9	**21 (6.0%)**
**Total**	**211**	**141**	**352**

### Quantitative results

#### Overall

Across all subscales (Scale 1–3) and the total score subscale, lower scores indicate less stigmatising attitudes. Total scores ranged from 19–51 for Imperial (n = 211) and 16–53 for NTU (n = 141); means and standard deviations are shown in Table 2. No significant difference in total scores were identified between the schools (p = 0.242).

**Fig. 1:**
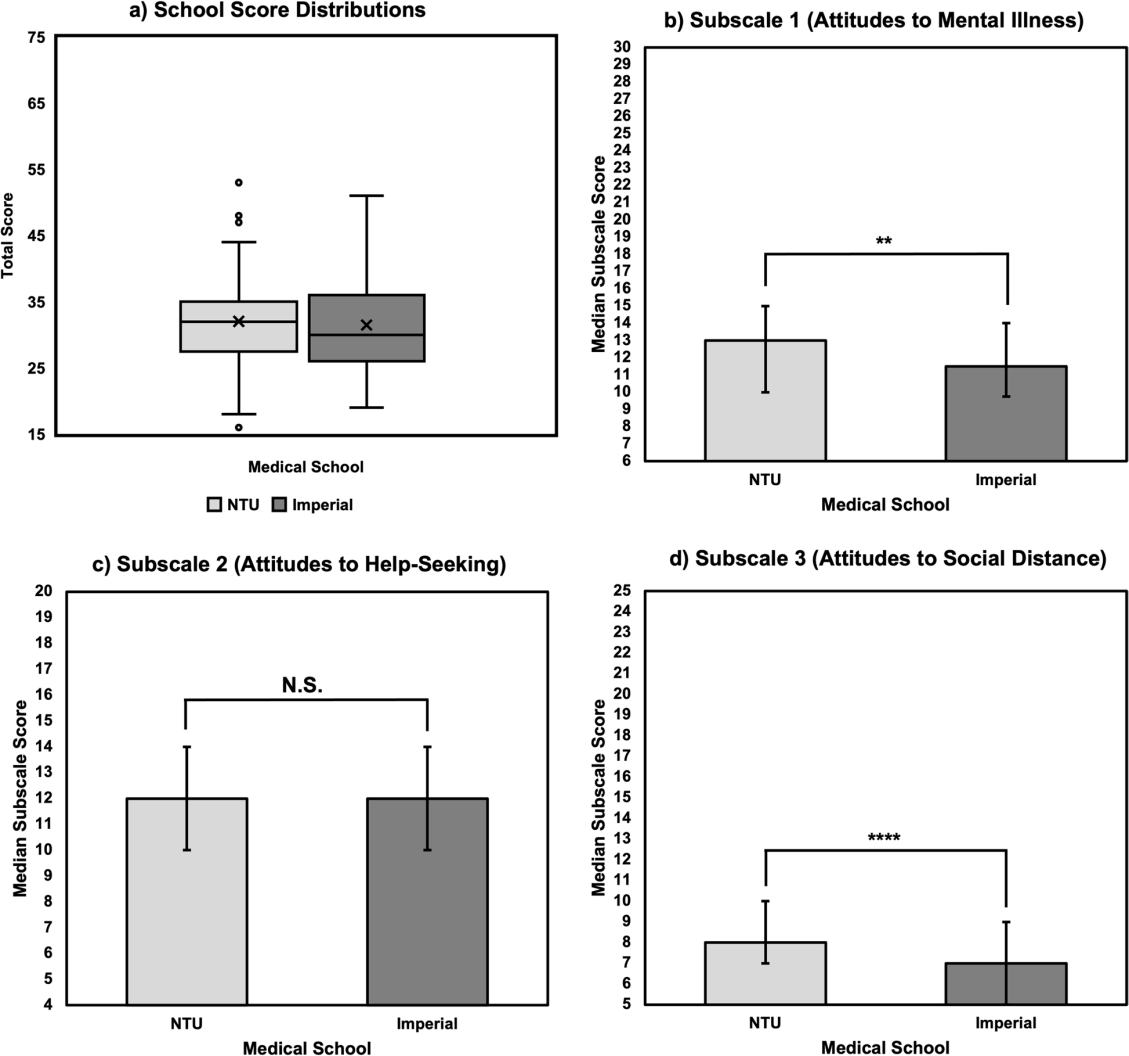
**a)** Box and whisker plot showing the total score distribution between the two schools (Imperial: n = 211, Median (M) = 30, Interquartile Range (IQR) = 10; NTU: n = 141, M = 32, IQR = 7). “X” represents mean (Imperial = 31.5; NTU = 32.0). No significant differences identified between schools when Mann–Whitney U test applied). **b)** Bar chart showing M ± IQR. Subscale 1 scores (Imperial: n = 211, 11.5 ± 4.25; NTU: n = 141, 13 ± 5). Error bars represent IQR. Significant difference identified when Mann–Whitney U test applied (p = 0.003). **c)** Bar chart showing M ± IQR. Subscale 2 scores (Imperial: n = 211, 12 ± 4; NTU: n = 141, 12 ± 4). Error bars represent IQR. No significant difference identified when Mann–Whitney test applied. **d)** Bar chart showing M ± IQR. Subscale 3 scores (Imperial: n = 211, 7 ± 4; NTU: n = 141, 8 ± 3). Error bars represent IQR. Significant difference identified when Mann–Whitney U test applied (p < 0.00001)

Small statistically significant differences were found in subscale analysis whereby NTU had higher stigma scores than Imperial for Subscales 1 (*Attitudes of healthcare providers towards people with mental illness)* (p = 0.003) and 3 *(Attitudes of healthcare providers towards social distance)* (p < 0.00001). No statistically significant differences were identified for Subscale 2 *(Attitudes of healthcare providers towards disclosure and help-seeking)* (p = 0.078)(Fig. 1).

#### Year group analysis

**Fig. 2:**
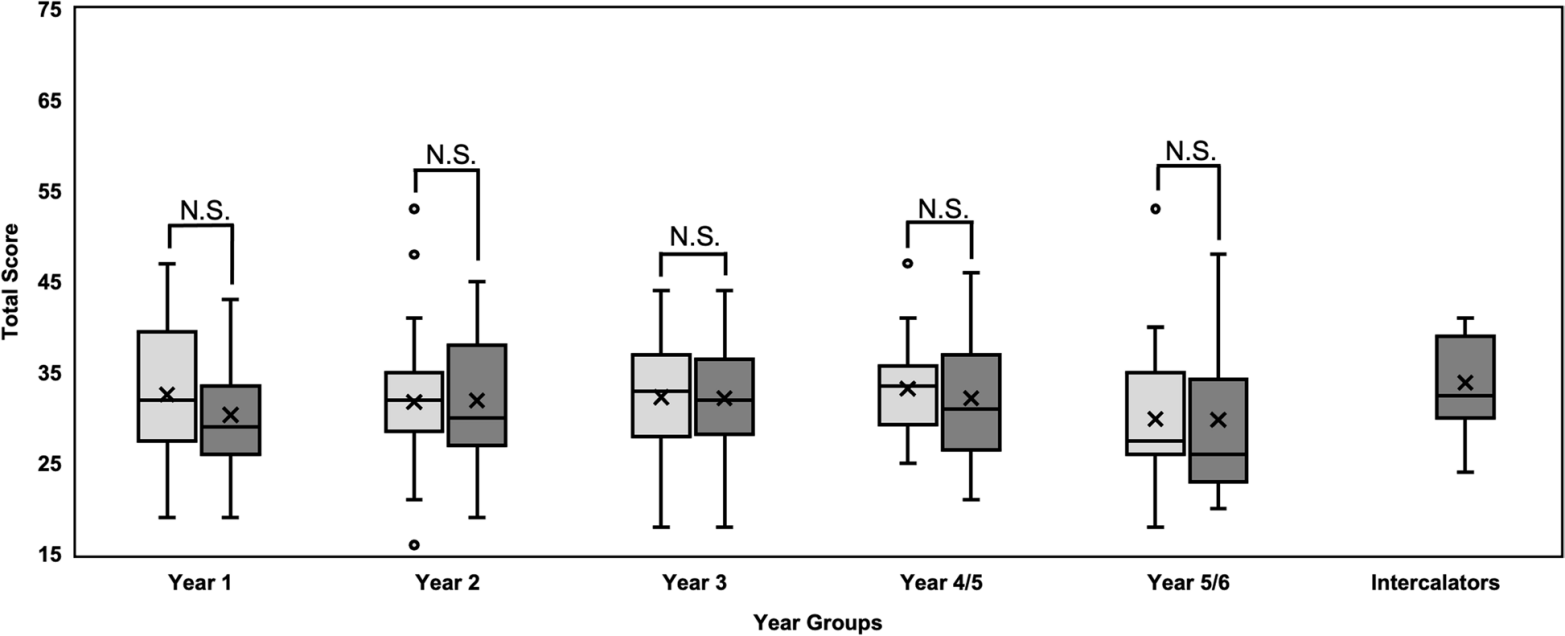
Box and whisker plots showing the distribution of total scores across year groups for both NTU and Imperial. “X” represents the mean. There was no significant difference for Year 1 (Imperial: n = 50, median (M) = 29, IQR = 7; NTU: n = 25, M = 32, IQR = 11), Year 2 (Imperial: n = 51, M = 30, IQR = 10.5; NTU: n = 49, M = 32, IQR = 6),Year 3 (Imperial: n = 29, M = 31.5, IQR = 9.5; NTU: n = 19, M = 33, IQR = 7.5), Year 4/5 (Imperial Year 5: n = 29, M = 31, IQR = 10; NTU Year 4: n = 28, M = 33.5, IQR = 5.5), or Year 5/6 (Imperial Year 6: n = 30, M = 26, IQR = 11; NTU Year 5: n = 20, M = 27.5, IQR = 9) when Mann–Whitney U tests were performed, with p > 0.05 in all instances

Years 1–3 at Imperial and NTU were compared directly with each other. However, Year 5 Imperial was compared with Year 4 NTU students and Year 6 Imperial was compared with Year 5 NTU students to account for intercalation at Imperial in Year 4. This ensured cohorts with similar amounts of clinically based education were compared to each other. No significant difference in total scores was observed between the medical schools for any single year group; distribution of year group scores can be compared in Fig. 2. Interestingly, Subscale 1 (*Attitudes of healthcare providers towards people with mental illness)* showed significant differences between the penultimate (p = 0.004) and final year (p = 0.011) medical student groups when comparing the two schools; Subscale 2 *(Attitudes of healthcare providers towards disclosure and help-seeking)* showed statistical differences between the Year 3 groups (p = 0.044) and Subscale 3 *(Attitudes of healthcare providers towards social distance)* showed statistical differences between each year group (Year 2: p = 0.019; Year 3: p = 0.012; Year 5/4: p = 0.016; Year 6/5: p = 0.078), except Year 1 (p = 0.226). Overall and subscale analyses are shown in Table 3.

**Table 3:** Table representing means, standard deviations (SD) (shown as mean ± standard deviation) and p-values for each school and year group comparison (p < 0.05) *Year 4 NTU maps to Year 5 Imperial; Year 5 NTU maps to Year 6 Imperial

	**Average Score** *(Mean* ± *Standard Deviation)*											
	**Overall Score** *(Range: 15–75)*			**Subscale 1 Attitudes to Mental Illness** *(Range: 6–30)*			**Subscale 2 Attitudes to Help-Seeking** *(Range: 4–20)*			**Subscale 3 Attitudes to Social Distance** *(Range: 5–25)*		
	**Imperial**	**NTU**	**p-value**	**Imperial**	**NTU**	**p-value**	**Imperial**	**NTU**	**p-value**	**Imperial**	**NTU**	**p-value**
**Overall** *(n* = *352)*	31.5 ± 6.5	32.0 ± 5.8	0.242	11.4 ± 3.2	12.6 ± 3.4	**0.003**	12.0 ± 3.1	12.6 ± 2.8	0.078	7.08 ± 2.4	8.37 ± 2.4	** < 0.00001**
**Year 1** *(n* = *75)*	30.3 ± 5.4	32.6 ± 6.9	0.160	11.3 ± 3.4	12.0 ± 4.3	0.444	12 ± 2.8	12.8 ± 3.0	0.287	7.08 ± 2.0	7.76 ± 2.3	0.226
**Year 2** *(n* = *100)*	32.0 ± 6.7	31.8 ± 6.2	0.620	11.8 ± 2.8	12.4 ± 3.3	0.418	12.4 ± 3.1	12.8 ± 3.1	0.555	7.41 ± 2.7	8.20 ± 2.1	**0.019**
**Year 3** *(n* = *48)*	31.9 ± 6.5	32.3 ± 6.2	0.820	10.8 ± 3.5	12.5 ± 3.7	0.271	11.3 ± 3.5	13.2 ± 2.5	**0.044**	6.86 ± 2.5	9.05 ± 2.8	**0.012**
**Intercalators** *(Imperial only: n* = *22)*	33.9 ± 5.3	N/A	N/A	12.1 ± 2.6	N/A	N/A	12.0 ± 2.6	N/A	N/A	6.77 ± 2.3	N/A	N/A
**Year 5/4** *(n* = *52)*	32.2 ± 6.9	33.3 ± 5.5	0.520	10.9 ± 2.9	13.0 ± 2.4	**0.004**	12.3 ± 3.7	12.1 ± 2.3	0.522	6.76 ± 2.4	8.25 ± 2.9	**0.016**
**Year 6/5** *(n* = *50)*	29.8 ± 8.2	29.9 ± 8.2	0.720	11.2 ± 3.6	13.6 ± 2.8	**0.011**	11.6 ± 2.8	12.1 ± 2.4	0.522	7.27 ± 2.5	9.05 ± 2.3	0.078

Kruskal–Wallis tests were performed to explore differences between year groups within the same medical school, for overall score and each subscale. No significant differences were found between year groups, suggesting stigma levels are unchanged throughout each individual medical school.

#### Qualitative results

A total of four major themes were identified from the interview analysis:

1. Perceptions of Medical Students Towards Mental Illness
2. Impact of Medical School Culture on Mental Health
3. University Support for Mental Health
4. Impact of Curriculum on Perceptions Towards Mental Health

Perceptions of medical students towards mental illness.

Students reported that mental illnesses were prevalent, particularly amongst themselves.

*“It's not uncommon that some students develop some psychiatric issues”* – Student 7 (Year 4), NTU

Interviewees felt that the general population, including themselves and peers, viewed those with a mental illness negatively, suggesting students’ perceptions are not meeting expectations.

*“To have some of these really problematic viewpoints still prevalent is really disheartening.”* – Student 1 (Year 1), Imperial

Varying opinions were seen regarding openness about mental health from those with mental illnesses, some suggesting that medical students tended to hide their mental illnesses.

*“I know that some people do seek help by themselves, but they do not involve any of their friends.”* – Student 6 (Year 3), NTU

Students also believed that mental illnesses were viewed more negatively compared to physical illnesses.

*“It also stems from a more Asian mindset. It's not a physical illness, right? Mental is all in your head.”* – Student 8 (Year 4), NTU

Impact of medical school culture on mental health.

Students believe the high-stress environment of medical school leads to poor mental health, considering factors such as competitiveness, general and academic culture.

*“Because of the competition, people’s depression and anxiety can get very bad.”* – Student 10 (Year 5), Imperial

There were fears of repercussions from being open with mental health experiences, particularly amongst those students from NTU, suggesting that it would stifle career progression.

*“[…] people don't seek help because of the fear of loss of employment in the future […] And that's not something that a school can fix.”* – Student 6 (Year 3), NTU

University support for mental health.

Students believed mental health support from universities was important, suggesting the positive impact faculty can have.

*“Faculty do a lot to show people that it's okay to come forward. They always signpost in newsletters […] they tried to de-stigmatise it.”* – Student 9 (Year 5), Imperial

*“Knowing that there's a house system and house tutor that we can talk to was very encouraging*.” – Student 3 (Year 2), NTU

They felt that universities considered mental health, however perceptions on support varied considerably. Those who believed the current support offered was sufficient complimented tutoring schemes, faculty attitudes, openness towards the topic and welfare and counselling support.

*“I feel like the college does well in providing support. So, we have counsellors, for example.”* – Student 3 (Year 2), Imperial

*“I will say that the people [Faculty] more on the ground are very well in tune with us.”* – Student 6 (Year 3), NTU

Those with negative perceptions commented on poor faculty and placement staff attitudes, the necessity for more skilled and representative tutors, and support groups.

*“Having personal tutors, or designated proper professionals, because our welfare team […] they’re not trained in anything.”* – Student 12 (Year 6), Imperial

*“The tutors are from a different generation*.” – Student 6 (Year 3), NTU

*“If there was improvement […] they could create peer support groups.”*– Student 7 (Year 4), NTU

They commented on a requirement for more regular meetings with their tutors to improve relationships and ability to share mental health problems.

*“If I speak to you once every 12 weeks over the phone for 10 minutes, I'm probably not going to confess that I'm depressed or anxious or whatever.”* – Student 12 (Year 6), Imperial

*“The tutors and faculty members can check on the students more frequently.”* – Student 7 (Year 4), NTU

Students agreed that sharing of mental health success stories from peers and faculty could be beneficial in opening talks around mental illness and enabling reduction in stigmatising attitudes throughout.

*“…Maybe open communication and open sharing from students and faculty?”* – Student 1 (Year 1), NTU

*“If they could find ways of getting people to come forward and talk about their experiences, I think that would allow people to feel more able talk about it.”* – Student 9 (Year 5), Imperial

Impact of curriculum on perceptions towards mental health.

Some students did not perceive much teaching on mental health within their curriculum.

*“From my experience of it, it's barely ever mentioned.”* – Student 4 (Year 2), Imperial

*“They haven't touched on mental illnesses at all in our curriculum.”* – Student 2 (Year 1), NTU

Other students felt that mental health was covered well within the curriculum, particularly in psychiatry-based placements.

*“I have a whole module in psychiatry. So, there is a lot of information that I get from there and a lot of knowledge that I draw upon.”* – Student 2 (Year 1), Imperial

These students complimented the curriculum, suggesting it exposed medical students to certain mental illnesses and in doing so helped shape perceptions and raise awareness, increasing empathy and understanding.

*“They have someone come in, […] saying what they've been through and explaining it and letting us ask questions, which I think was really good.”* – Student 8 (Year 4), NTU

*“I've been taught a lot more about […] what things you should be looking out for in people with mental health problems.”* – Student 7 (Year 4), Imperial

Students identified a need to improve the curriculum for its inability to address negative perceptions, suggesting requirements for earlier inclusion and increased teaching, particularly within holistic care.

*“They could emphasise […] there are genetic components, stressor components, more than just telling you about the criteria in the DSM-5.”* – Student 6 (Year 3), NTU

*“It would be quite useful to have people who speak about how their religion has helped or this culture has helped […] Rather than it still being quite so blanket treatment.”* – Student 5 (Year 3), Imperial

*“I think it does need to be addressed earlier.”* – Student 3 (Year 2), Imperial

There was also acknowledgement that curricular changes would be hard to initiate, due to the dense nature of the syllabus.

*“I also understand that the curriculum is quite intensive. And that it is already quite packed as it is, even with the normal schedule, there's not much time in-between.”* – Student 7 (Year 4), NTU

## Discussion

Our study demonstrated a significant level of stigma held by medical students at both Imperial and NTU, affirming the necessity for further elucidation of drivers of this, as the global mental health burden is projected to rise over the next decade [4]. Thus, there is renewed impetus for evaluating recommendations aiming to mitigate such stigma.

No significant differences in the overall presence of stigmatising attitudes between medical students at Imperial and NTU were seen. There were no overall differences across year groups within each school. However, NTU showed statistically more negative attitudes for Subscales 1 *(Attitudes of healthcare providers towards people with mental illness)* and 3 *(Attitudes of health care providers towards social distance)* compared to Imperial. Subscales 1 and 3 relate to the role of the stigmatiser and not the stigmatised (Subscale 2), suggesting intersectional factors as per the MISF, such as Singaporean culture or race, contribute to stigmatising attitudes, as corroborated by interview responses and other literature [29]. Our qualitative responses indicate that students felt their medical schools had reasonable systems to support students’ mental wellbeing, such as with tutoring and house systems. This may suggest why there was no overall difference in Subscale 2.

The scores obtained in this study can be interpreted by benchmarking against other studies across the world, such as in Saudi Arabia [30] and Malaysia [31], using the same OMS-HC-15 scale [27]. The similarity between Singapore and the UK is possibly explained by the intersectional factor of socioeconomic similarity between the two countries relative to others [15], or may reflect improved perceptions over time, as shown by Lien et al. [32]. Another consideration is the increased incidence of mental illness in many countries because of the COVID-19 pandemic [33, 34] improving understanding of mental illness and thus contributing to the lower stigma scores evidenced in our study.

Whilst both schools’ scores lie in the mid-lower segment of the scale, a degree of stigma remains when considering the minimum possible score of 15. Similarly, stigmatising attitudes remain prevalent among students worldwide as shown by Babicki et al. [15]. This residual stigma may be due to the self-stigma experienced by medical students because of the high-stress, competitive nature of medical school inherently promoting poor mental health, as well as concerns of career progression, as discussed in our qualitative analysis and other studies [35]. Notably, Fox et al*.* [17] have used the MISF to highlight a paucity of research focusing on the impact of self-stigma, or the role of the stigmatised, as opposed to that of the stigmatiser. Nonetheless, a 2.5 total score reduction was seen between a 2017 Singaporean study on medical students and our present-day NTU score [36], suggesting that there remains some scope for perceptions to improve. Of note, the 2017 study did not include one question in their final calculation of overall score. Its average overall score would be higher had this question been included, suggesting an even greater improvement over the years than is evident at first glance [36].

This improvement in score compared to 2017 may be due to differences in curriculum, as other studies have shown that curriculum directly influences stigma [37, 38]. This could further explain why Imperial and NTU had similar overall scores given their related curricula comparative to markedly different scores amongst schools in other countries. Contrary to other studies [16, 38, 39], there were no statistical differences across year groups within each school, suggesting that no singular year’s curriculum at Imperial or NTU is currently addressing stigmatising attitudes within its cohort, even when considering the new Imperial curriculum. The lack of meaningful difference between earlier and later years contrasted qualitative responses from both schools which often cited the benefit of the psychiatry clinical attachment in their penultimate year, and the relative lack of other learning opportunities. The continuation of stigma throughout the year groups could be related to the stereotypical nature of which medical conditions are taught to help students identify and recognise these characteristics in patients. Stereotypes are one of the underpinning factors contributing to stigmatisers’ attitudes within the MISF.

### Limitations

Survey response rate at Imperial (10.0%) was relatively lower than NTU’s response rate (19.3%) and may confer a degree of nonresponse bias that limits the generalisability of results somewhat. Moreover, the skew of gender differed between NTU and Imperial, with NTU showing a preference for male respondents (male = 54%, female = 46%), in keeping with demographic data at the time (male = 62%, female = 38%) [40], and Imperial favoured female respondents (male = 37%, female = 63%), which was similarly reflected in their 2019–2020 demographic data (male = 46%, female = 54%) [41]. Analyses, however, showed gender was not a significant factor in influencing stigma score. Two-tailed unpaired t-tests found no significant difference between males and females at Imperial (p = 0.77), nor at NTU (p = 0.70). Moreover, a Mann–Whitney U test found no significant overall difference between males and females at both medical schools (p = 0.37, U = 13,469). We lack further publicly accessible data on the demographics of the two medical schools, so it is not possible to compare respondents to non-respondents, and hence we are unable to evaluate nonresponse bias in this instance.

Survey distribution timing (October 2021) failed to consider if and when senior years had undertaken their psychiatric placement, therefore affecting year group analyses and assessment of longer-term stigma reduction.

Schwenk et al. [42] showed that medical students declaring a mental illness had less-stigmatising attitudes compared to those who do not. However, in this study, too few students from NTU declared mental ill health and declaration of diagnoses were minimal, disallowing for meaningful analysis within these groups. Imperial survey respondents notably had a higher proportion with a history of mental ill health (37.4%) compared to NTU respondents (12.8%) which may have been a confounding factor. Demographic factors were not considered when selecting interview participants.

Imperial students in year 3 and below were following a different curriculum studied by students in Years 4–6 which may have impacted on the lack of difference across years at Imperial.

Whilst the OMS-HC-15 scale is a useful validated measure it remains unclear how such numerical scores translate to clinical practice.

### Recommendations

A longitudinal evaluation of if and how progression through medical school alters attitudes is warranted. This is of particular importance at Imperial to assess the efficacy of the redesigned curriculum.

The implementation of opportunities where faculty/peers share success stories of their own mental health journeys could address the negative perceptions shown in Subscale 2 (*Attitudes of healthcare providers towards disclosure and help-seeking)/*self-stigma of the stigmatised per the MISF. This has been found to have a successful impact in other institutions [43]. Qualitative responses focussing on improvements to university services included increasing training for staff in pastoral roles and increasing diversity amongst such staff that they better represent the student body, with more regular tutor check-ins so students feel more empowered to be open sharing problems with them. This would be aimed at addressing students’ own mental health, through use of pastoral tutoring and counselling services, and should be applied to further address students’ help-seeking behaviours per Subscale 2 and self-stigma.

Expanding psychoeducation within curricula to give a wider overview of conditions, including epidemiological features, the course and progression of illnesses, as well as expansion on social and holistic approaches to treatment could be beneficial in addressing the stigmatiser’s attitudes towards mental illness, as suggested in our qualitative results. Other suggestions include earlier inclusions within the curriculum. Different media of delivery such as educational videos, increased patient cases and stories and specific workshops focussing on these aspects may help reinforce these messages. These methods have been proven to be effective in addressing overall stigmatising attitudes, specifically those of Subscales 1 *(Attitudes of healthcare providers towards people with mental illness)* and 3 *(Attitudes of health care providers towards social distance),* in previous studies [44]*.*

## Conclusions

This paper has identified and gauged the presence of mental health stigma in two medical schools of differing cultures. Underlying causes of stigma were further explored and recommendations provided to address these perceptions utilising qualitative views from students themselves, whilst applying the Mental Illness Stigma Framework. Stigma scores were similar between Imperial and NTU but lower relative to other countries, perhaps accounted for by intersectional factors or differences in curriculum. Suggested improvements from students include improvements to student welfare services and sharing of peer/faculty struggles with mental health to improve self-stigma and help-seeking behaviours. The mental health curriculum should be introduced earlier into students’ medical education and adopt a more holistic approach through a broader variety of teaching mediums.

### Supplementary Information

**Additional file 1. **Opening minds survey for health care providers.

**Additional file 2. **Interview questions.

## Data Availability

The datasets used and/or analysed during the current study are available from the corresponding author on reasonable request.

## Abbreviations

HCP: Health care provider
Imperial: Imperial college London
MISF: Mental illness stigma framework
NTU: Nanyang technological university
OMS-HC-15: Opening minds stigma scale for healthcare providers
UK: United Kingdom
