# Supplementary material for: Medical student perceptions of mental illness: a cross-sectional transnational study in two medical schools
Source: BMC Med Educ. 2023 Dec 20;23:981. doi: 10.1186/s12909-023-04962-2 (PMC10731839; doi:10.1186/s12909-023-04962-2)
Supplement: Supplementary file 1 — Additional file 1. Opening minds survey for health care providers. [file 12909_2023_4962_MOESM1_ESM.pdf]

OMS-HC15Pre

Opening Minds Survey for Health Care Providers

	Strongly Disagree	Disagree	Neither Agree nor Disagree	Agree	Strongly Agree
1. I am more comfortable helping a person who has a physical illness than I am helping a person who has a mental illness. (Original 1)	☐	☐	☐	☐	☐
2. If a colleague with whom I work told me they had a managed mental illness, I would be just as willing to work with him/her. (Original 3) **R**	☐	☐	☐	☐	☐
3. If I were under treatment for a mental illness I would not disclose this to any of my colleagues. (Original 4)	☐	☐	☐	☐	☐
4. I would see myself as weak if I had a mental illness and could not fix it myself. (Original 6)	☐	☐	☐	☐	☐
5. I would be reluctant to seek help if I had a mental illness. (Original 7)	☐	☐	☐	☐	☐
6. Employers should hire a person with a managed mental illness if he/she is the best person for the job. (Original 8) **R**	☐	☐	☐	☐	☐
7. I would still go to a physician if I knew that the physician had been treated for a mental illness.(Original 9) **R**	☐	☐	☐	☐	☐
8. If I had a mental illness, I would tell my friends. (Original 10) **R**	☐	☐	☐	☐	☐
9. Despite my professional beliefs, I have negative reactions towards people who have mental illness. (Original 12)	☐	☐	☐	☐	☐
10. There is little I can do to help people with mental illness. (Original 13)	☐	☐	☐	☐	☐
11. More than half of people with mental illness don't try hard enough to get better. (Original 14)	☐	☐	☐	☐	☐
12. I would not want a person with a mental illness, even if it were appropriately managed, to work with children. (Original 17)	☐	☐	☐	☐	☐
13. Healthcare providers do not need to be advocates for people with mental illness. (Original 18)	☐	☐	☐	☐	☐
14. I would not mind if a person with a mental illness lived next door to me. (Original 19) **R**	☐	☐	☐	☐	☐
15. I struggle to feel compassion for a person with mental illness. (Original 20)	☐	☐	☐	☐	☐

Coding

On the 15-item scale: items 2, 6, 7, 8, 14 require reverse scoring.

Outcomes:

Total OMS-HC 15 mean score

Total scale scores could range from 15 to 75 and a lower score indicates less stigma

Subscales:

1. **Attitudes of health care providers towards people with mental illness (5 items = 1, 9, 10, 11, 13, 15)**
   The concepts captured in this subscale relate to general attitudes towards people with mental illness and the role of health care providers

2. **Attitudes of health care providers towards disclosure and help-seeking (4 items = 3, 4, 5, 8)**
   The concepts captured in this subscale relate to self-disclosure of mental illness and help seeking behavior

3. **Attitudes of health care providers towards social distance (5 items = 2, 6, 7, 12, 14)**
   The concepts captured in this subscale relate to a willingness to readily engage persons with mental illness in various activities and relationships
