# Supplementary material for: Medical student perceptions of mental illness: a cross-sectional transnational study in two medical schools
Source: BMC Med Educ. 2023 Dec 20;23:981. doi: 10.1186/s12909-023-04962-2 (PMC10731839; doi:10.1186/s12909-023-04962-2)
Supplement: Supplementary file 2 — Additional file 2. Interview questions. [file 12909_2023_4962_MOESM2_ESM.docx]

**Interview Questions**

Perception

1. What is your mental image of a person with mental illness
2. How would you describe someone with poor mental health
3. How is mental illness represented within the media within your country? What impact do you believe this has?
4. What is the general perception of mental illness in your country?
   1. What is the general perception of mental illness in your medical school?
   2. How do you think your family members/friends view mental illness?
5. How do you think the perception of mental illness in your country differs from other countries?

Factors Affecting Perception

1. What factors have shaped your own views of mental illness?
   1. How has medical school shaped your views of mental illness, if at all?
   2. Are there any cultural factors involved?
2. In our questionnaire, respondents that have identified as gender fluid/non-heterosexual have a lower stigma towards mental illness. What are your thoughts about that

What Can be Done

1. How well do you think your medical school addresses mental illness?
   1. What can be done to reduce stigmatisation of others, perhaps in the curriculum and the medical school?
